# Special Issue “Molecular Insights into the Developmental Origins of Health and Disease”

**DOI:** 10.3390/ijms262311579

**Published:** 2025-11-29

**Authors:** Paramjit S. Tappia, Bram Ramjiawan

**Affiliations:** 1 Asper Clinical Research Institute, St. Boniface Hospital, Winnipeg, MB R2H 2A6, Canada; bramjiawan@sbrc.ca; 2 Department of Pharmacology and Therapeutics, Max Rady College of Medicine, Rady Faculty of Health Sciences, University of Manitoba, Winnipeg, MB R3E 0T6, Canada

There is now a wealth of epidemiological, clinical, and experimental evidence that have concluded that the risk of developing chronic, noncommunicable diseases in adulthood may be influenced by molecular and genetic aspects [1,2,3,4,5], as well as lifestyle and environmental experiences during early life [6,7,8,9,10,11,12,13]. Fetal development and infancy are characterized by the rapid growth, development, and maturation of organ systems. It has now become increasingly evident that several pathophysiological conditions, including diabetes and cardiovascular disease, that occur in adolescence and adulthood, may have their origins during fetal or postnatal development. While maternal nutrition (poor quality and quantity) is the most examined aspect in terms of influencing the development of fetal organ systems, paternal stressors have also emerged as critical developmental and molecular elements that can increase offspring’s predisposition to adverse health outcomes in later life.

Dr. David Barker first published findings proposing a direct link between prenatal nutrition and late-onset coronary heart disease in *The Lancet* [14]. This was the first paper to propose the core idea of the Barker hypothesis. From that time, the Developmental Origins of Health and Disease (DOHaD) framework has advanced significantly in recent years, driven by new mechanistic insights, improved analytical tools, and an expanded understanding of how early-life environments shape long-term health trajectories. These developments are strengthening the evidence base for early prevention and reinforcing the need for integrated life-course approaches to public health.

Rapid growth in epigenomic, transcriptomic, and metabolomic technologies has enabled precise mapping of how early exposures regulate gene expression and organ development. High-resolution epigenetic analyses now identify stable molecular signatures associated with maternal diet, metabolic health, stress, and environmental toxicants [15,16,17,18,19,20,21,22,23,24]. Single-cell approaches have further clarified how these exposures influence the developmental programming of key tissues, particularly through the placenta, which is now recognized as an active mediator of environmental signals.

Recent work highlights the central role of the maternal–infant microbiome axis in shaping immune function and metabolic homeostasis. Alterations in maternal microbial ecology, linked to nutrition, antibiotic use, or stress, have measurable effects on neonatal microbiome establishment and downstream health outcomes [25,26,27,28,29,30]. Parallel advances have also established that paternal factors contribute meaningfully to early programming. Epigenetic and small-RNA profiles in sperm reflect paternal nutritional and environmental exposures, challenging the maternal-centric paradigm that long dominated the field [31,32].

DOHaD research is moving beyond association toward targeted intervention. Trials addressing maternal nutrition, gestational diabetes, physical activity, and stress reduction demonstrate measurable benefits in offspring metabolic and neurodevelopmental outcomes. These findings underscore early development as a critical window of plasticity, where modifying environmental and social conditions can produce lasting health effects.

The field has increasingly acknowledged the influence of social determinants including inequity, historical trauma, and environmental disadvantage on early programming. Research conducted in partnership with marginalized communities has been particularly important in illustrating how intergenerational stress and structural conditions become biologically embedded [33,34,35]. These perspectives emphasize the necessity of culturally grounded, community-led approaches in DOHaD research and policy translation.

With this background and perspectives, this Special Issue provides a glimpse into the influential role of internal and external factors on fetal genetics, biochemistry and physiology. Eight outstanding papers, from experts in the field, provide a broad range of contributions detailing advances in the field of developmental biology and the reinforcement of a life-course model of health that prioritizes preconception care, maternal well-being, and early childhood development as essential components of chronic disease prevention in later life.

We earlier analyzed the phospholipid profile of the developing heart of rats exposed to low-protein (LP) diet in pregnancy [36]. It was found that maternal LP diet can induce changes in the phospholipid profile and fatty acid content of the developing heart, which may have implications for metabolism of the neonatal heart. After 25 years, the topic of cell membrane lipid composition and its influence on cellular structural integrity and functionality has been reviewed from a developmental and pathological perspective by Ali and Szabó [37]. In this review, the authors summarize the diversity of membrane lipids and their constituent fatty acids in healthy organisms and the essential roles they play in cellular function and highlight the functional significance of membrane-associated fatty acids and their influence on key cellular physiological responses.

Early life exposures to environmental/maternal chemicals can exert negative effects on the developing fetus [38]. There are three articles that demonstrate this. In the review by Tain and Hsu [39], various environmental chemicals to which pregnant mothers are commonly exposed can disrupt fetal programming, leading to a wide range of cardiovascular–kidney–metabolic phenotypes. The authors present that the aryl hydrocarbon receptor (AHR) plays a key role as a ligand-activated transcription factor in sensing these environmental chemicals that can increase the predisposition to cardiovascular diseases, hypertension, diabetes, obesity, kidney disease, and non-alcoholic fatty liver disease. The authors emphasize the importance of circumventing toxic chemical exposure during pregnancy and extend the understanding of AHR signaling, which may potentially lessen the global burden of CKM syndrome.

In the original research paper by Strunz et al. [40], the impact of polybrominated diphenyl ethers (PBDEs), commonly used as synthetic flame retardants, and they are present in a variety of products, including electronics, polyurethane foams, textiles, and building materials, on weight gain and insulin insensitivity in offspring has been examined. In a mouse model, these researchers demonstrated that maternal exposure to BDE-47 results in weight gain in female offspring together with an impaired glucose and insulin tolerance in both female and male mice. It was also found that this chemical toxin increased adipogenesis as well as neuronal dysregulation of energy homeostasis, attributed to impaired leptin signaling. The authors concluded that these mechanistic aspects may contribute to the observed weight gain as well as impaired insulin and glucose tolerance.

ETV6::RUNX1-positive pediatric acute lymphoblastic leukemia is frequently considered to have a prenatal origin. In an interventional cohort study (1221 pregnancies), the impact of maternal corticosteroid use during pregnancy and its association with this form of leukemia at birth are examined. In this study by Benítez et al. [41], it was observed that corticosteroid use for lung maturation during pregnancy was significantly associated with ETV6::RUNX1 in 39 neonates, particularly if applied before 26 weeks of gestation or if betamethasone was used. Thus, it was concluded that prenatal exposure to corticosteroids within a critical time window could increase the risk of developing ETV6::RUNX1+ preleukemic clones and potentially leukemia after birth. In addition, it was stated that modulation of ETV6::RUNX1 preleukemia may potentially prevent this subtype of childhood leukemia.

The following two articles shed some light on the developmental mechanisms that can influence brain function and lung morphogenesis. KCC2 (Potassium–Chloride Cotransporter 2) and NKCC1 (Sodium–Potassium–Chloride Cotransporter 1) are crucial for maintaining chloride ion balance inside and outside neurons, playing complementary roles in the regulation of GABAergic (gamma-aminobutyric acid) inhibition and chloride homeostasis in the nervous system. Brain-derived neurotrophic factor (BDNF) influences the functioning of these co-transporters and normal brain function. Their dysregulation is implicated in neurological disorders. In the study by Hamze et al. [42], it was reported that proBDNF delays the GABA shift polarity, thereby maintaining neurons in an immature state, which could be linked to the behavioral deficits in electroporated rats. These actions carry significant implications for cognitive processes and neural circuitry, providing insights into the intricate interplay between neurotrophic factors and neuronal functions. The authors concluded that these findings advance the understanding of neurodevelopmental processes that could potentially lead to the development of targeted therapies for brain disorders.

It is known that pulmonary branches are formed during the early stages of embryonic lung development through an intricate process known as lung branching morphogenesis that is influenced by retinoic acid (RA). Fernandes-Silva et al. [43] explored the role of RA as a metabolic modulator in an ex vivo model of lung explant culture of embryonic chicken lungs. It was revealed that RA signaling stimulation redirects glucose towards pyruvate and succinate production rather than to alanine or lactate. Of note, the inhibition of RA signal transduction reduced lung branching, which resulted in a cystic-like phenotype while promoting mitochondrial function. Accordingly, the authors suggested that RA is a regulator of tissue proliferation and that it plays an important role in determining lung metabolism during branching morphogenesis. Taken together, such information adds to the understanding of lung development and cystic-related lung disorders.

By using a rat model where maternal CKD is induced with adenine, Tain et al. [44] have demonstrated that the offspring develop high blood pressure in adulthood. These researchers also tested whether citrulline, a non-essential amino acid that can boost the production of nitric oxide (NO) and exhibits antioxidant properties, could prevent this outcome. Citrulline effectively normalized blood pressure in the offspring of CKD mothers. Its protective effects were associated with improved NO signaling, reduced renal (pro)renin receptor expression, and induced beneficial shifts in gut microbiota. Overall, perinatal citrulline supplementation enhanced NO availability and prevented hypertension programmed by maternal CKD. The study underscores the importance of maternal nutrition for fetal development but also shows how specific nutrients could be used in conditions where there is an adverse environmental experience that could predispose the offspring to chronic disease in later life.

In the last paper of this Special Issue by Lee et al. [45], an interesting topic of external pathogens, liquid biopsy, and biomarkers that could be predictive of later systemic disease is highlighted. Tick bites and related illnesses are increasing, but current diagnostic tests detect only well-known tick-borne pathogens and cannot explain poorly defined conditions like Australia’s debilitating symptom complexes attributed to ticks (DSCATT). This study investigated whether blood samples could capture molecular signals originating from tick-bitten skin, offering a less invasive way to monitor local biological responses. By comparing multi-omics profiles from skin biopsies at the bite site with matched peripheral blood samples, researchers found that the pathways involved in extracellular matrix organization and platelet degranulation were activated in the skin within 72 h of a tick bite. Importantly, these same signals appeared in the blood and remained elevated for up to three months. These findings are suggestive that systemic blood profiles can reflect local tissue events after a tick bite, supporting the potential of blood-based “liquid biopsies” for future diagnostic and mechanistic studies on the development of systemic illnesses that may be tick-borne pathogen-based.

In conclusion, the last decade has marked a period of remarkable progress in DOHaD research. Technological advancements, integrative multi-omics, a broader appreciation of paternal and sociocultural influences, and the emergence of intervention studies are reshaping the field. What once seemed a revolutionary idea that early life shapes lifelong health is now an actionable framework guiding clinical practice, public health strategy, and global policy. The next advancement is clear: moving from understanding mechanisms to implementing solutions. The promise of DOHaD lies not only in explaining disease origins but in enabling societies to build healthier futures by protecting and empowering the earliest stages of life.

We extend our sincere appreciation to all contributors, each highly regarded in their respective fields, for helping to create this exceptional Special Issue. We hope that both experts and readers with a broader interest in DOHaD will find the content informative and thought-provoking. In addition, we hope that it will stimulate, motivate, and inspire biomedical scientists and researchers into further inquiry in order to advance our understanding of the essential role played by diverse maternal, paternal, and environmental factors in fetal biology and predisposition to adverse health conditions. Furthermore, this Special Issue will serve as a beneficial resource for medical students and fellows as well as graduate students that have a desire to learn about DOHaD.
